# Involvement of Glutamate Transporter-1 in Neuroprotection against Global Brain Ischemia-Reperfusion Injury Induced by Postconditioning in Rats

**DOI:** 10.3390/ijms11114407

**Published:** 2010-11-03

**Authors:** Weiqiao Zhang, Yifeng Miao, Sanquan Zhou, Baofeng Wang, Qizhong Luo, Yongming Qiu

**Affiliations:** 1 Department of Neurosurgery, Ren Ji Hospital, Shanghai Jiao Tong University School of Medicine, Shanghai 200127, China; E-Mails: zhang951154@sina.com (W.Z.); 553909021@qq.com(Y.M.);kingwww629@126.com (B.W.); 13958354209@139.com (Q.L.); 2 Department of Neurosurgery, YuYao People’s Hospital, Zhejiang Province, 315400, China; E-Mail: zsq8989@yahoo.com (S.Z.)

**Keywords:** ischemic postconditioning, brain ischemia reperfusion injury, neuroprotection, glutamate transporter 1(GLT-1)

## Abstract

Ischemic postconditioning refers to several transient reperfusion and ischemia cycles after an ischemic event and before a long duration of reperfusion. The procedure produces neuroprotective effects. The mechanisms underlying these neuroprotective effects are poorly understood. In this study, we found that most neurons in the CA1 region died after 10 minutes of ischemia and is followed by 72 hours of reperfusion. However, brain ischemic postconditioning (six cycles of 10 s/10 s reperfusion/re-occlusion) significantly reduced neuronal death. Significant up-regulation of Glutamate transporter-1 was found after 3, 6, 24, 72 hours of reperfusion. The present study showed that ischemic postconditioning decreases cell death and that upregulation of GLT-1 expression may play an important role on this effect.

## 1. Introduction

Ischemic stroke is a leading cause of mortality and adult morbidity in humans. Extensive research has been conducted to find drugs that reduce injury following brain ischemia/reperfusion; however, no effective neuroprotective drugs have been found for use in clinical practice [1].

Ischemic postconditioning refers to several transient reperfusion and ischemia cycles after an ischemic event and before a long duration of reperfusion. Its cerebral protective effects have been proven in different animal models [2–5], but its mechanism has not been clarified.

Glutamate is one of the main neurotransmitters and works at a variety of excitatory synapses in the nervous system [6]. However, it is also believed to lead to glutamate excitotoxicity—an important mechanism of cell death induced by ischemic insult [7]. Many studies have proven that the extracellular concentration of glutamate is elevated to levels leading to neuronal death in ischemia.

Glutamate transporters are essential for maintaining low concentration by removing the glutamate from the extracellular space. At present, five glutamate transporters have been identified in the rat brain. These glutamate transporters, are GLAST, GLT-1, EAAT3, EAAT4 and EAAT5 [8].

Glutamate transporter-1 (GLT-1), which is the most abundant glutamate transporter in brain, plays a principal role in keeping the glutamate concentration low by removing the glutamate released at the synapse [9]. In view of the importance of GLT-1 in the maintenance of appropriate glutamate concentrations, it is reasonable to infer that GLT-1 is involved in the protective effect of brain ischemic postconditioning. However, there have been no reports regarding the role of GLT-1 in brain ischemic tolerance generated by ischemic postconditioning.

In this study, we tested the hypothesis that ischemic postconditioning reduces neuronal death by upregulating GLT-1. We used the 4-vessel occlusion (4-VO) animal model, Hematoxylin/erosin and cresyl violet staining were used to elucidate the protective effect of postconditioning in the hippocampal CA1 at 72 hours after reperfusion and hippocampal GLT-1 expression was measured at 3, 6, 24, 72 hours of reperfusion to demonstrate the neuronal protective mechanism of ischemic postconditioning.

## 2. Results and Discussion

### 2.1. Neuropathological Evaluation of Hippocampal CA1 by Hematoxylin/Erosin Staining

**Figure 1.**
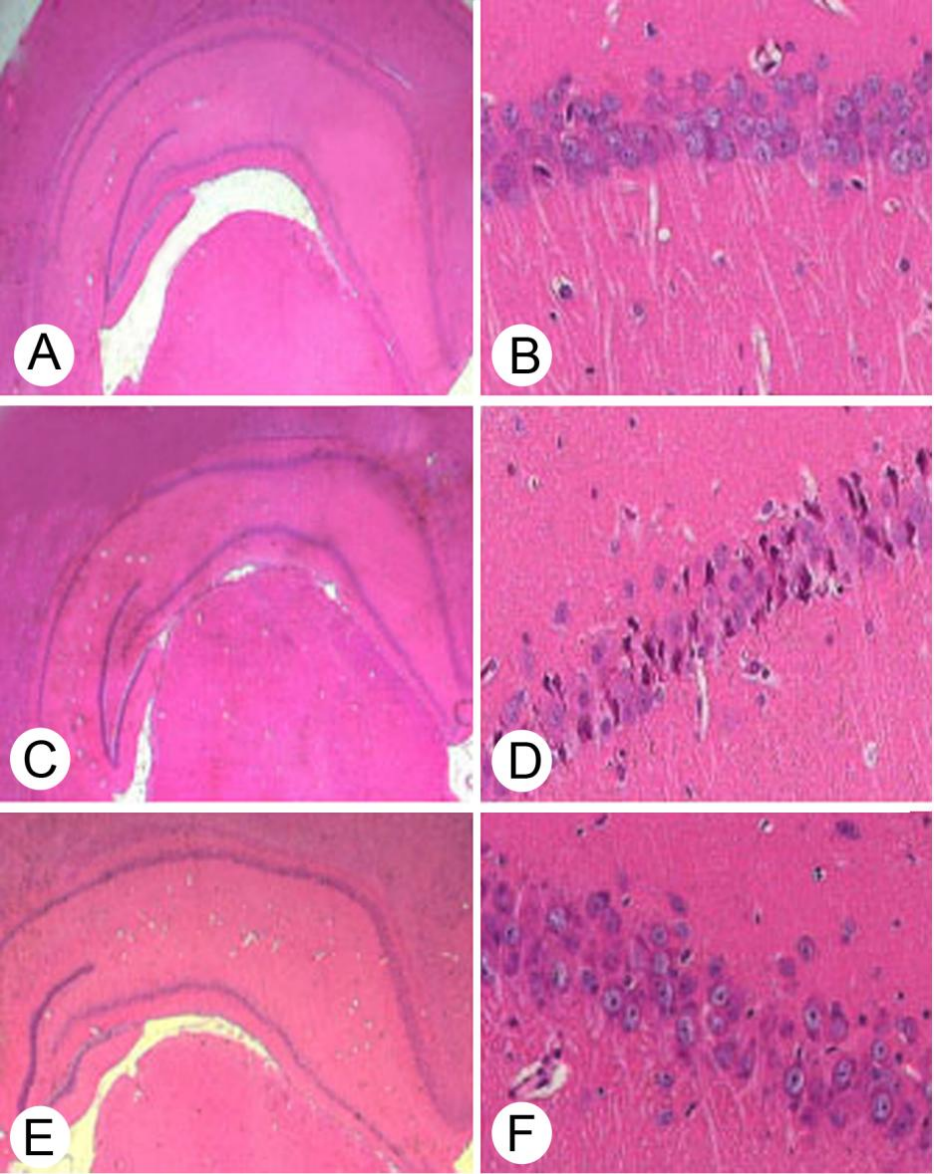
Neuropathological evaluation of hippocampal CA1 by hematoxylin and eosin staining. Representative photomicrographs in the hippocampal region (×40) (A, C, E) and hippocampus CA1 (×400) (B, D, F).

The distribution of pyramidal cells in CA1 was examined 72 hours after reperfusion. As shown in Figure 1, in the sham group, pyramidal neurons were layered and in regular arrangements with large, round, transparent, and intact nuclei (Figure 1A, 1B). In the I/R group, the neurons in the CA1 region were severely damaged, in disorderly arrays, significantly reduced in number, and characterized by pyknotic and indistinct nuclei (Figure 1C, 1D). Six cycles of 10-second/10-second of reperfusion/re-occlusion significantly decreased cell death in CA1 (Figure 1E, 1F).

### 2.2. Neuron Counts in Hippocampal CA1, as Determined by Cresyl Violet Staining

**Figure 2.**
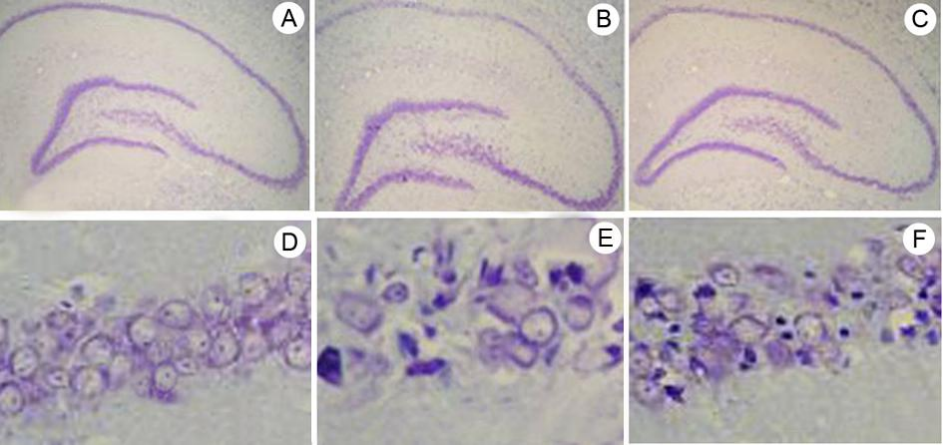
Neuroprotective effect of ischemic postconditioning on neuron death induced by global ischemia/reperfusion in the hippocampal CA1, as determined using cresyl violet staining. A, D: sham group; B, E: ischemia/reperfusion group; C, F: Ischemic postconditioning group. Magnification, (×40) (A, B, C) and (×400) (D, E, F).

**Figure 3.**
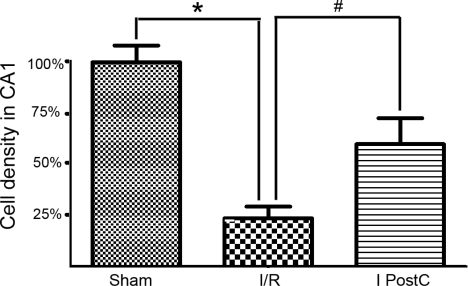
Histogram representing numbers of surviving neurons in CA1/mm after 72 hours of reperfusion (n = 4). *: I/R group *versus* Sham group (*P* < 0.01). #: I PostC group *versus* I/R group (*P* < 0.01).

We used the cell density to assess the effect of ischemic postconditioning in the hippocampal CA1 region by using cresyl violet staining (Figure 2). In the sham group, pyramidal cells displayed round, pale, stained nuclei. We observed that 72 hours after re-perfusion, most CA1 pyramidal cells were destroyed in I/R group. The number of surviving neurons increased significantly in the I PostC group as compared to the I/R group (*P* < 0.01). The numbers of surviving pyramidal cells in the hippocampal CA1 region of the I/R and I PostC groups, respectively, were ∼23.5% and ∼62.8% percent of those observed in rats treated with the sham operation (Figure 3).

### 2.3. Expression of GLT-1

**Figure 4.**
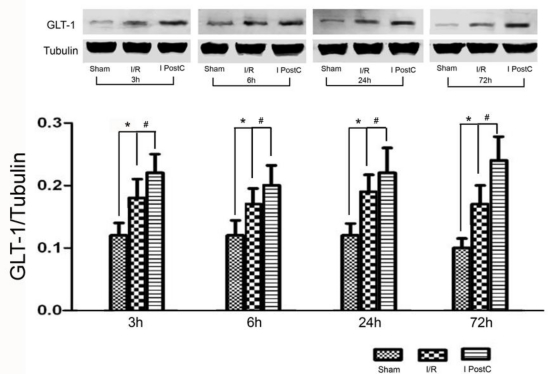
Expression of GLT-1 protein at 3, 6, 24 and 72 hours after reperfusion. Hippocampus in the sham group, I/R group and I PostC group. *: GLT-1 of I/R group increased significantly as compared with that of the sham group (*P* < 0.01). #: I PostC group had greater upregulation of GLT-1 compared to the I/R group at every time point (*P* < 0.05).

Western blot analysis was carried out to identify the expression of GLT-1 at the protein level in the hippocampus (Figure 4). The fact that GLT-1 was expressed in sham-treated animals indicated its presence in the normal rat hippocampus. At each designated time point after reperfusion, the amount of GLT-1 in both the I/R and I PostC groups increased significantly as compared with that of the sham group (*P* < 0.01). We observed an increase in GLT-1 protein in the I PostC group as compared with the I/R group at 3 hours, 6 hours, 24 hours and 72 hours after reperfusion, respectively. (*P* < 0.05).

### 2.4. Discussion

Our study demonstrated that after six cycles of 10 s/10 s ischemic postconditioning administered to global cerebral ischemic rats followed with reperfusion for 72 hours, the extent of neuronal cell loss was decreased significantly in the ischemic injury-prone hippocampal CA1 region. This finding suggests that ischemic postconditioning can protect neurons. We found that ischemic postconditioning can increase the GLT-1 level in hippocampus. This may be one of the mechanisms of ischemic postconditioning. Due to the upregulation of GLT-1, it is possible to decrease glutamate accumulation in the synaptic cleft and thereby reduce the overactivation of postsynaptic glutamate receptors, thus attenuating the death of neurons.

Ischemic postconditioning (I PostC) is carried out by administering several cycles of short reperfusion/ischemia at the early stage of reperfusion. Many researchers have proven that I PostC protects the brain and have been trying to study the mechanism from different perspectives. Using 4-VO global cerebral ischemic rats, Wang *et al.* found that I PostC can reduce neuronal death in hippocampus and increase the spatial memory of rats. This may be related to I PostC’s ability to improve disturbed cerebral blood flow and inhibit the release of cytochrome c [3]. Using MCAO rats, Xing *et al.* found that I PostC can reduce infarct size, increase neurologic score, upregulate Bcl-2 and Hsp70, and inhibit caspase-3, Bax and cytochrome c. The authors speculated that brain protection by I PostC may be due to inhibition of cell apoptosis [2]. Gao *et al.* found that the effects of I PostC are related to changes in the Akt, MAPK and PKC signaling pathways [10,11].

The excitotoxicity of glutamate is considered to be an important reason for neuronal death after ischemia [12–14]. Therefore, reducing the amount of glutamate in extracellular fluid during ischemia would lessen the damage to neurons. Glutamate is reabsorbed mainly by glutamate transporters. Although neurons express glutamate transporter, it is usually believed that glutamate transporters, especially GLT-1, play the dominant role in maintaining extracellular glutamate level [9,15].

GLT-1 could be involved in many neuropathic processes. For example, in studies on cerebral ischemia, some authors have presumed that the decrease of GLT-1 is the reason for increases in the level of glutamate [16–19]. In other studies, it was found that levels of glutamate transporter increased after ischemia. The authors considered this as a compensatory response to increased glutamate reabsorbed under these conditions [20,21]. In addition, it has been shown that GLT-1 is also involved in pathological processes such as brain trauma, neurodegenerative disease and epilepsy [22–25]. Namura *et al.* found that downregulation of GLT-1 can enhance brain edema after ischemia [26]. Our previous work showed that GLT-1 may play a role in ischemia-related epilepsy [27].

The role of GLT-1 in I PostC has not yet been studied. In this study, we found that hippocampal GLT-1 in I/R group increased compared to the sham group. After I PostC, a further increase of GLT-1 was found. Thus, we speculate that the upregulation of GLT-1 may be a self-protection mechanism after cerebral ischemia; I PostC can enhance the protective effect, thereby reducing neuronal damage.

## 3. Experimental Section

### 3.1. Animal Groups

All animal protocols were approved by the School of Medicine Animal Care and Use Committee of Shanghai Jiao Tong University. Eighty-four adult male Sprague-Dawley rats (220–250 g) were used for this study. The rats were randomly assigned to three groups: sham (Sham) group (3 hours, n = 6; 6 hours, n = 6; 24 hours, n = 6; 72 hours, n = 10); ischemia/reperfusion (I/R) group (3 hours, n=6; 6 hours, n = 6; 24 hours, n = 6; 72 hours, n = 10) and ischemic postconditioning (I PostC) group (3 hours, n = 6; 6 hours, n = 6; 24 hours, n = 6; 72 hours, n = 10). Animals were housed in a 12 hours/12 hours light/dark cycle environment with free access to water and food.

### 3.2. Animal Model and Surgical Procedure

The 4-vessel occlusion (4-VO) animal model, which we have described previously, was used to establish global brain ischemia [28]. In brief, rats were anesthetized by intraperitoneal administration of 20% chloral hydrate (350 mg/kg). Both vertebral arteries were electrocauterized and the bilateral common carotid arteries were separated on day 1. On the following day, the bilateral common carotid arteries were clamped with aneurysm clips for 10 minutes to cause global brain ischemia. Rats that had lost the righting reflex, had dilated pupils, and were unresponsive to light, were used in the experiments.

In the sham group, rats were anesthetized, both vertebral arteries were electrocauterized and the bilateral common carotid arteries were separated, but not occluded. In the ischemia/reperfusion group, rats were subjected to 10 minutes of global ischemia and then the aneurysm clip was loosened to permit reperfusion. In the ischemic postconditioning group, rats were subjected to 10 minutes of global ischemia, which was followed by ischemic postconditioning.

**Figure 5.**
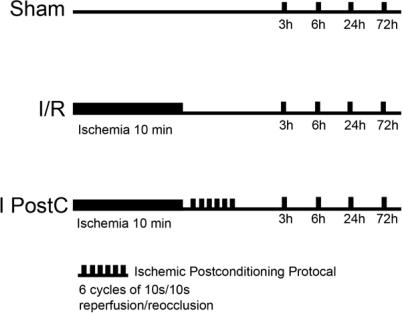
Experimental protocol used to determine the effects of ischemic postconditioning (I PostC) after ischemia/reperfusion. Sham: sham-operated rats, rats were anesthetized, both vertebral arteries were electrocauterized and the bilateral common carotid arteries were separated, but not occluded; I/R: rats subjected to 10 minutes of 4-vessel occlusion (4-VO) followed by reperfusion; I PostC, rats treated with ischemic postconditioning after 10 minutes of 4-VO by six cycles of 10 s/10 s reperfusion/ischemia (at the beginning of reperfusion). Reperfusion lasted for 3, 6, 24 or 72 hours.

The ischemic postconditioning protocol was as follows: rats in the I PostC group were subjected to six cycles of 10 seconds of reperfusion and 10 seconds of ischemia by removing aneurysm clips and then occluding the clips (Figure 5).

### 3.3. Histological Assessment

At 72 hours after reperfusion, rats (n = 4 in each group) were deeply anesthetized with chloral hydrate and perfused with normal saline, followed by perfusion with 4% paraformaldehyde. The brains were quickly removed and postfixed in 4% paraformaldehyde for 24 hours. The postfixed brains were embedded in paraffin. Coronal sections 5 μm thick were cut with a microtome. The sections were stained with Hematoxylin/erosin and cresyl violet, and examined with a light microscope. The cell density among hippocampal CA1, which was expressed as the ratio of survive cells per 1 mm length in CA1 as counted under a light microscope, was used to assess the extent of brain damage.

### 3.4. Western Blotting

Western blotting was used to measure levels of GLT-1 protein in each animal in each group at all time points examined (3, 6, 24, and 72 hours after reperfusion; n = 6). The hippocampus was homogenized for 30 min at 4 °C with an ultrasonic wave in 200 mL of an RIPA lysis buffer containing: 50 mM Tris-HCl pH 7.4, 150 mM NaCl, 1 mM EDTA, 1% Triton x-100, 1% Sodium Deoxycholate, 0.1% SDS, add 1 mM PMSF and proteintase inhibitor cocktail. The homogenates were then lysed in SDS buffer. Protein concentration was determined using the Bio-Rad Protein Assay Kit and the sample (20 μg) was separated on Tris-glycine gel, then transferred to a nitrocellulose membrane, and immunoblotted. Blots were probed with rabbit polyclonal anti-GLT-1 (1:1000; Abcam). The blot was then incubated with IRDye 800CW goat anti-mouse secondary antibody (1:4000, LI-COR Biosciences, Lincoln, NE, U.S.) and bound antibodies were detected using the LI-COR imaging system. The blots were stripped and reprobed with mouse anti-tubulin (1:1000; Sigma) as a loading control. The experiments were repeated three times to obtain an average value. The band density values were calculated as a ratio of GLT-1/tublin.

### 3.5. Statistical Analysis

Data are presented as mean ± SEM. Data analysis was performed using SAS software. Statistical significance was determined by one-way ANOVA with a Newman-Keuls post test for neuron counting. Western blot analysis was performed using two-way analysis of variance (ANOVA) followed by the Student-Newman-Keuls test. Values were considered to be significant when *P* < 0.05.

## 4. Conclusions

Our study is the first to demonstrate that GLT-1 levels increased after ischemic postconditioning, which helps to further demonstrate the protective effect of I PostC on brain.
